# Biofilm formation by *Streptococcus mutans* and its inhibition by green tea extracts

**DOI:** 10.1186/s13568-021-01232-6

**Published:** 2021-05-25

**Authors:** Sara Moataz Zayed, Mohammad Mabrouk Aboulwafa, Abdelgawad Mohamed Hashem, Sarra Ebrahim Saleh

**Affiliations:** 1 Medical Sciences Department, Faculty of Dentistry, The British University in Egypt, Cairo, Egypt; 2 Microbiology and Immunology Department, Faculty of Pharmacy, Ain Shams University, Cairo, Egypt; 3 Faculty of Pharmacy, King Salman International University, South Sinai, Ras-Sedr, Egypt; 4 Microbiology and Immunology Department, Faculty of Pharmacy, The British University in Egypt, Cairo, Egypt

**Keywords:** *Streptococcus mutans*, Dental caries, Antibiofilm activity, Green tea extracts

## Abstract

Dental Caries is considered one of the most existing and worldwide common diseases related to the oral cavity affecting both children and adults. *Streptococcus mutans* is the main cariogenic microorganism involved in the dental caries progression. Natural products such as herbal plants were found to have less side effects and economic value than those of the chemically synthesized antibiofilm agents. This study aimed to isolate *Streptococcus mutans* from different oral samples taken from saliva and dental plaques specimens to determine their capability for biofilm formation and to evaluate the antibiofilm activity of aqueous and alcoholic green tea extracts. The results revealed that 35, 4 and 1% of recovered dental plaque isolates exhibited strong, moderate and weak biofilm formation capabilities versus 26, 12 and 2% for those recovered from saliva. Two green tea extracts (aqueous and alcoholic) were tested for their antibiofilm formation activity against some selected *S. mutans* isolates. The results showed that the minimum biofilm inhibitory concentrations (MBICs) of the alcoholic and aqueous green tea extracts were in the range of 3.1 to 12.5 mg/ml and 6.5 to 50 mg/ml, respectively. Accordingly, green tea extracts can be incorporated in various oral preparations for preventing dental caries.

## Introduction

Oral biofilm is three-dimensional complex structure of different microorganisms inhabiting the oral cavity; if remained for long time without treatment or intervention, the biofilm can undergo maturation leading to dental caries development. The presence of microorganisms in biofilm structure of exopolysaccharide matrix form enhances  their pathogenicity as it improves their resistance to the immune system of the host and different antimicrobial agents (Gurenlian 2015). Dental caries development is associated with biofilm formation affecting large population worldwide. It is believed that the bacteria of species *Streptococcus mutans* is the primary etiologic agent participating in this serious condition. *S. mutans* plays an essential role in forming multi-dimensional and complex structure on the oral mucosa and tooth enamel (Wang et al. 2020). It acquires some cariogenic properties such as its ability to adhere to solid surfaces, colonize the oral cavity and the ability to survive the acidic condition of the oral cavity (Krzyściak et al. 2014). Also, *S. mutans* uses the carbohydrate producing acidic metabolites which leads to acidic destruction and demineralization of the tooth enamel removing the mineral materials and thus inducing dental caries. From the virulence factors of *S. mutans* that contribute to oral biofilm development is its ability to produce glucosyltransferase B, an extracellular enzyme which is responsible for glucan formation from sucrose contained in diet. The synthesized glucan causes the adhesion of *S. mutans* to the tooth enamel and other microorganisms to each other that increases protection against mechanical host-clearance forces and different antimicrobial agents (Ito et al. 2020). The essential requirements for biofilm formation involved in dental caries development related to *S. mutans* are the protein bacterium interactions which involve the sucrose dependent mechanism and the sucrose independent mechanism. The sucrose dependent mechanism mainly depends on glycosyltransferases (GTFB, GTFC and GTFD) produced by *S. mutans* which are responsible for synthesis of glucan from sucrose. The synthesized sticky nature of glucan provides adhesion of bacteria to each other and cohesion of bacteria to tooth enamel, provides adherence with saliva proteins in pellicle and resistance to clearance by mechanical host forces. While the sucrose independent adhesion is initiated by interaction of salivary agglutinins and *S. mutans* with the surface associated protein P1 (also known as I/II antigen, SpaP or Pac1. Also, several factors have significant role on biofilm production related to *S. mutans* such as carbohydrate intake and metabolism, aciduricity and acidogenicity. The progression of dental caries is mainly correlated to the diet content of carbohydrate and frequency of their consumption. Sucrose is the most cariogenic carbohydrate as it constitutes the main metabolism for *S. mutans*. Carbohydrates are mainly taken up by two transporters, phosphoenol pyruvate sugar phosphotransferase system and ATP binding cassette transporters. *S. mutans* metabolizes carbohydrates to form biofilm and to adhere the microorganisms to each other and to the tooth enamel allowing the pathogen to tolerate different physiological factors such as nutrient availability, aerobic to anaerobic conditions and pH changes. In addition, *S. mutans* has the ability to mount and adapt response to low pH due to ATPase translocating proton contributing in aciduricity, the lower the pH at which ATPase can function as the metabolic end products builds up, the more competitive the microorganism in biofilm structure. *S. mutans* is one of the most acidogenic microorganism found in biofilm because it can produce acids from the fermented carbohydrates other than any oral streptococci. In addition, *S. mutans* produces mutacins (bacteriocins) which is important factor in colonization of *S. mutans* in dental biofilm (Banu 2010; Matsumoto-Nakano 2018; Krzyściak et al. 2014).

The currently used chemical antibiofilm agents such as quaternary ammonium salts, iodine compounds and fluoride can lead to various side effects (Rolim et al. 2019; Mehrishi et al. 2020). Also, the overdose and long administration of antimicrobial agents can lead to antimicrobial resistance which is considered a serious health issue nowadays (Razuqi et al. 2012; Fajriani et al. 2020). So, in order to overcome virulence biofilm characters, there has been a shift towards using natural herbal plants that could be used for their possible effects as antibiofilm agents. These natural products have the advantages of being less costly and of lower side effects. Green tea (*Camellia sinensis)* has been proposed in this study to be evaluated and tested for its antibiofilm activity. Green tea contains four major flavonoids which are catechins epicatechin (EC), epigallocatechin (EGC), epicatechin gallate (ECG) and epigallocatechin gallate (EGCG) (Fajriani et al. 2020). EGCG plays a significant role in inhibiting biofilm development and progression as it targets the glucosyltransferase enzymes responsible for converting sucrose contained in diet to glucan, the building block of the exopolysaccharide matrix (Hengge 2019).

The aim of this study was to investigate the biofilm formation capability of *S. mutans* isolates collected from dental plaque and saliva specimens and as natural products, two green extracts were used for evaluation of their antibiofilm activity against biofilm former *S. mutans* isolates.

## Materials and methods

### Microorganisms

A total of 150 isolates were recovered from seventy five dental plaque samples (dp) and seventy five saliva samples (ss) collected from patients having different degrees of dental caries. The samples were obtained from the British University in Egypt (BUE). The clinical specimens of the strongest biofilm producers were deposited at the culture collection of Ain Shams University under the codes of (CCASU-SM 25) and (CCASU-SM 26). The standard bacterial strain *Streptococcus mutans* (ATCC 25175) was obtained from Faculty of Dentistry, Cairo university**.** These strains were subcultured routinely on brain heart infusion agar and mitis salivarius medium for maintenance and stored as glycerol stock at −80 ℃ for long term preservation.

### Chemicals

The chemicals used in this study included: glacial acetic acid, glycerol and saline obtained from El-Nasr Chemicals Co., crystal violet powder obtained from Sigma- Aldrich, methanol obtained from El Gomhouria Co. and chlorhexidine obtained from Arab Drug Company, Cairo, Egypt.

### Media and kits

The used culture media included: brain heart agar, the product of Lab M Ltd., Topley House, Bury, Lanarkshire, United Kingdom; brain heart infusion broth, the product of Oxoid, USA from which two different concentrations of sucrose solution were prepared (4% and 2%); mitis salivarius medium, the product of HIMEDIA, India. API 20 Strep kit used for identification of recovered clinical isolates was the product of BioMérieux.

### Recovery and identification of *Streptococcus* species isolates from collected saliva and dental plaques specimens

Saliva and dental plaque specimens (75 each) collected from patients suffering from dental caries were used for recovery of *Streptococcus* species.One loopful from each sample was streaked over plate of mitis salivarius medium and incubated at 37 °C for 48 h (Acumedia 2011). The colonies showing the distinct characters of *Streptococcus mutans* (hard raised, convex, undulate, opaque, pale blue colonies and frosty glass appearance) were picked up and purified on the same medium (Al-mudallal et al. 2008). The purified isolates were identified by commercial biochemical test system API 20 Strep following the manufacturer’s instructions. For identification by API 20 Strep, the inoculum was prepared by adding several loopfuls from the pure fresh culture of the test isolate to 2 ml ampoule of API suspension medium to form a dense suspension (4 McFarland). The cupules and tubules of the API Strep were inoculated by the resultant bacterial suspension following the inoculum sizes mentioned by the manufacturer. A drop of mineral oil was added to each cupule for some tests requiring anaerobic conditions. Then the lid was placed on the tray. The inoculated strips were then incubated at 37 °C and the results were read at the time intervals stated by the manufacturer. For long term preservation, glycerol stock cultures were prepared from single purified colonies of the identified isolates which were then kept at −80°.

### Testing biofilm formation by the recovered isolates

#### Bacterial inoculum preparation

A pure single colony of each test isolate as well as the standard reference *Streptococcus mutans* were used for inoculation of 3 ml aliquots contained in wassermann tubes of brain heart infusion broth supplemented with 2% sucrose. Bacterial cultures were then incubated at 37 °C for 48 h.

### By test tube method (TM)

The bacterial cultures grown in the wassermann tubes previously prepared were decanted and the tubes were washed gently three times with sterile saline, then dried at room temperature. Three ml crystal violet solution (0.1%) were added to each wassermann tube, then the tubes were left at room temperature for 15 min, followed by decantation of crystal violet solution and three times gentle washing with deionized water. Biofilm formation was detected by the appearance of visible violet colour on the walls and the bottoms of the test tubes. The tubes were photographed and the tested isolates were categorized as zero, absent; 1, weak; 2, moderate; 3, strong biofilm producers (Al-mudallal et al. 2008; Mathur et al. 2006).

### By microtiter plate method (MTP)

The culture prepared before was adjusted to 0.5 McFarland using brain heart infusion broth, then diluted 100 fold in the same medium containing 2% sucrose. The diluted culture of each test isolate as well as the standard reference strain were distributed in the wells of 96 flat bottom microtiter plate at 200 µl per well (3 wells for each isolate). The plates were incubated at 37 °C for 48 h, then the plates were processed as follows: The bacterial culture was decanted by inversion of microtiter plate and the plates were washed three to four times with sterile saline (0.9%). Aliquots (200 µl each) of methanol were added to the washed wells and the plates were left at room temperature for 20 min followed by decantation of the fixative methanol and leaving the plates to get dry. The plates were stained by adding 200 µl of 0.1% crystal violet to each well and left for 15 min followed by three times washing with distilled water and the plates were then left at room temperature to get dry while inverted. The adherent cells with their formed biofilms (if any) were resolubilized by adding 200 µl of 33% glacial acetic acid to each well and the OD was then measured at 600 nm using microplate reader. Each clinical isolate was tested in triplicate, the data was then averaged and standard deviation was estimated. The mean OD value obtained for control wells (containing non inoculated media) was deducted from the average OD values of each test isolate (Kwasny and Opperman 2010). The results obtained were used to classify the test isolates according to their biofilm formation capabilities as shown in Table 1.

**Table 1:** OD (OD600 nm) scale levels used for classification of the tested isolates according to their biofilm formation capabilities (Mohamed et al. 2013)

OD value	Biofilm formation
OD ≤ 2* OD_c_^a^	Weak biofilm producer
2*OD_c_ ≤ OD ≤ 4*OD_c_	Moderate biofilm producer
4*OD_c_ ≤ OD	Strong biofilm producer

^a^OD_c_ is OD of the control wells

### Determination of antibiofilm activity of aqueous and alcoholic green tea extracts using microtiter plate assay

Ten isolates recovered from dental plaque specimens and other 10 recovered from saliva samples were used for testing the antibiofilm activity of aqueous and alcoholic green tea extracts. Each 10 isolates comprised of 2 (weak), 2 (moderate), and 4 (strong) biofilm producers.

### Preparation of aqueous green tea extract

Aqueous green tea extract was prepared by boiling 10 g grinded green tea leaves **(***Camellia sinensis****)*** obtained from a herbal medicine store for 5 min in 100 ml distilled water followed by filtering the cold extract through Whatman filter paper number 1. The filtrate was then dried at 80 °C in an incubator and the paste obtained was about 300 mg (Kwasny and Opperman 2010; Faraz et al. 2012). The paste obtained was reconstituted in distilled water to 100 mg/ml.

### Preparation of alcoholic green tea extract

This was carried out similar to the aqueous green tea extract preparation described before except that 50 ml methanol was used instead of 100 of distilled water. The paste obtained was dissolved in distilled water to a concentration of 100 mg/ml (Kwasny and Opperman 2010; Faraz et al. 2012).

### Determination of antibiofilm activity using microtiter plate assay

The 48 h brain heart culture of each test clinical isolate was adjusted to 0.5 McFarland using sterile brain heart infusion broth (1.5 × 10^8^). The adjusted suspension for each test isolate was further diluted 100 fold using brain heart infusion broth (represented the isolate inoculum suspension). Aliquots of 100 µl of single strength brain heart infusion broth were distributed in the wells of 96 microtiter plate expect the first column of the plate which contained 100 µl double strength brain heart infusion broth in each well. Aliquots of 100 µl of green tea extract (100 mg/ml) were transferred to the first well of each row, then two fold serial dilution was carried out up to the tenth columns from which 100 µl aliquots were discarded. The last 2 columns (11th and 12th) represent 2 controls, one control was for biofilm formation level of untreated bacterial isolates and the second one represented the biofilm inhibition caused by 0.2% chlorohexidine. Aliquots (100 µl) from the inoculum suspensions of the test isolates were added to wells of three rows assigned to each test isolate. To check sterility of medium, aliquots of 100 µl of brain heart infusion broth containing either 2 or 4% sucrose were added to two separate rows. The inoculated plates were incubated at 37 °C for 48 h and the procedures were completed as described before. For each test isolate, the percent inhibition of biofilm formation caused by green tea extract (aqueous or alcoholic) was calculated using the following formula

$$\left[ {1 - \left( {{\text{average O}}{{\text{D}}_{{\text{600 }}}}{\text{of treated isolate/average O}}{{\text{D}}_{{\text{600 }}}}{\text{of untreated isolate }}} \right)} \right] \times 100$$

For the tested agent, the minimum biofilm inhibitory concentration (MBIC) is determined by identifying the lowest concentration of the used agent that inhibited biofilm formation ≥ 80% (Kwasny and Opperman 2010).

## Results

### Isolation and purification of *Streptococcus mutans* from saliva and dental plaque specimens

A total number of 150 bacterial isolates including 75 isolates from saliva and other 75 from dental plaque collected specimens were recovered on mitis salivarius medium. Single colony from each clinical isolate was subcultured on the same medium for further purification and preparation of stock culture for long term preservation at −80 °C. The saliva sample isolates were given codes from ss1 to ss75 while dental plague isolates were given codes from dp1 to dp75.

### Identification of the collected clinical isolates

**Fig. 1:**
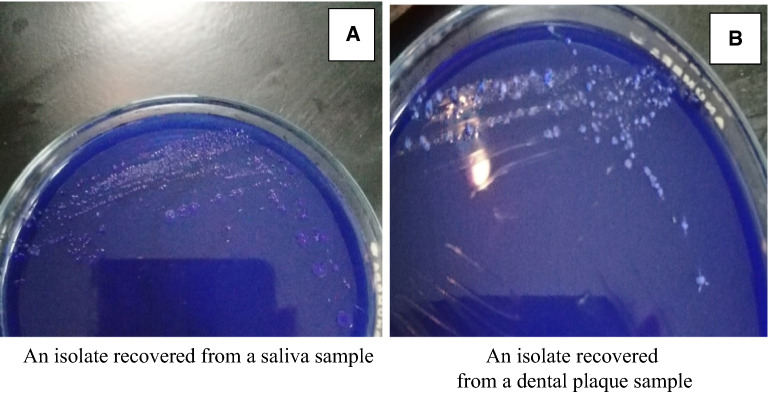
Colonies’ appearance of two representative isolates recovered from a saliva (**A**) and a dental plaque sample (**B**) on mitis salivarius medium

Gram staining of the purified clinical isolates were done for preliminary identification, the test isolates showed Gram positive cocci arranged in chains. Isolates were also characterized depending on their colonial shape on mitis salivarius medium. Those suspected to be *Streptococcus* species showed distinct hard raised, convex, undulate, opaque, pale blue colonies and frosty glass appearance as shown in Fig. 1.

The collected isolates were further checked for their identity using commercial biochemical test system 20600 API 20 strep (BioMérieux) as *Streptococcus mutans* (100 isolates) and *Streptococcus salivarius* (50 isolates). The most common characteristic reactions for both *Streptococcus* species (*mutans* and *salivarius*) are represented in Table 2.

**Table 2:** Common biochemical characteristic reactions for *S. mutans* and *S. salivarius* as determined by API 20 Strep

Test (Substrate/Reaction/Enzyme)	Result	
	*S. mutans*	*S. salivarius*
VP (Acetoin production)	+	+
Hydrolysis (HIPpuric acid)	−	−
ESC (ß- glucosidase hydrolysis)	+	+
PYRA (pyrrolidinyl arylamidase)	−	−
αGAL (α-Galactosidase)	+	−
ßGUR(ß-Glucuronidase)	−	−
ßGAL (ß-Galactosidase)	−	−
PAL (Alkaline phosphatase)	−	+
LAP (Leucine aminopeptidase)	+	+
ADH (Arginine dihydrolase)	−	−
RIB (d-Ribose)	−	−
ARA (l-Arabinose)	−	−
MAN (d-Mannitol)	+	−
SOR (d-Sorbitol)	+	−
LAC (d-Lactose)	+	−
TRE (d-Trehalose)	+	+
INU (Inulin)	+	−
RAF (d- Raffinose)	+	+
AMD (Starch)	−	+
GLYG (Glycogen)	−	−

### Detection of biofilm formation by the collected *Streptococcus* isolates

#### As determined by tube method (TM)

**Fig. 2:**
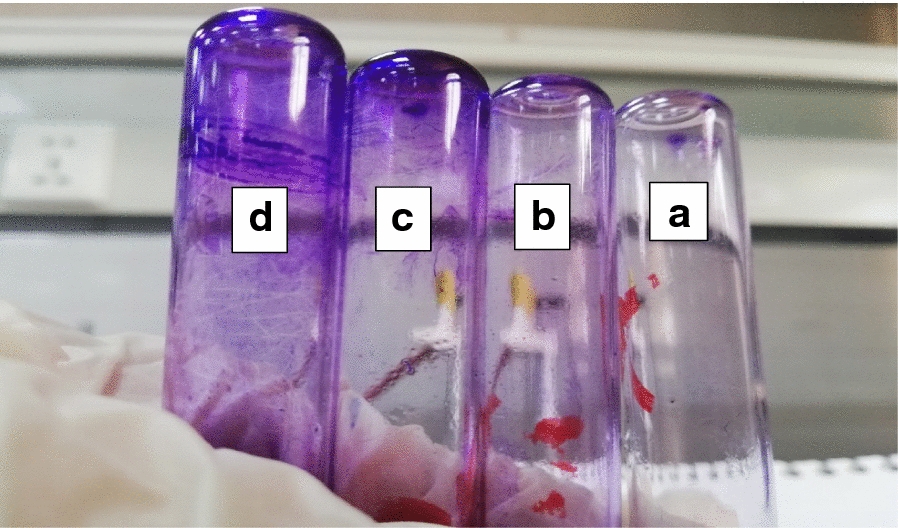
Violet color developed by some *Streptococcus mutans* isolates showing different degrees of their formed biofilms as determined by test tube method. (**a**) non biofilm producer, (**b**) weak biofilm producer, (**c**) moderate biofilm producer and (**d**) strong biofilm producer

**Fig. 3:**
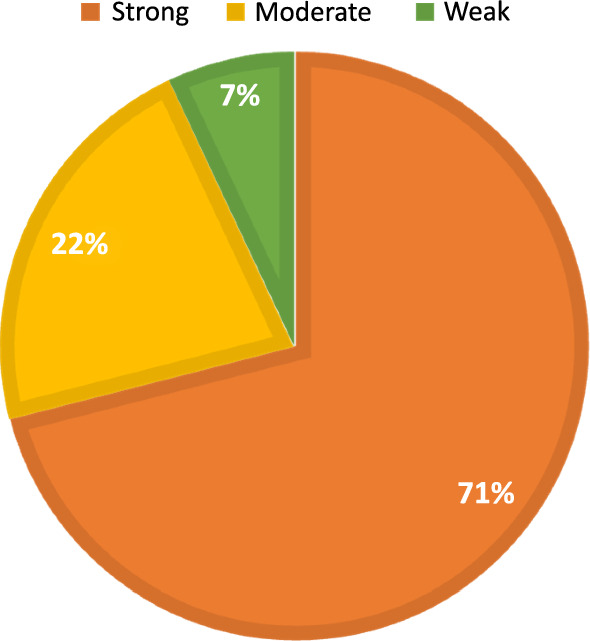
Relative percentages of *S. mutans* isolates showing weak, moderate, and strong biofilms

The results of tube method (TM) revealed that out of total 150 clinical specimens recovered from oral cavity, 100 isolates were recognized as biofilm producers while the rest of isolates exhibited no biofilm formation capabilities. The presence of violet visible film lining the walls and bottoms of the Wassermann tubes indicates biofilm production and Fig. 2 showed the degree of biofilm formed by some representative isolates. Among the biofilm producers, 7, 22 and 71 isolates were considered as weak, moderate and strong biofilm producers, respectively as shown in Fig. 3.

### As determined by microtiter plate method (MTP)

**Fig. 4:**
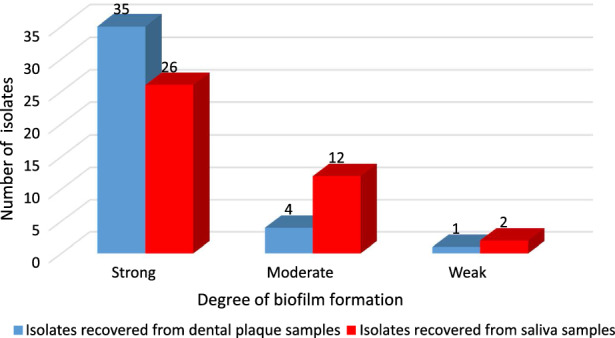
Relative number of isolates showing strong, moderate, and weak biofilm formation of *Streptococcus mutans* test isolates recovered from dental plaque and saliva samples

The results of microtiter plate (MTP) assay showed that out of 100 *Streptococcus mutans* test isolates only 80 test isolates form biofilm, half of these isolates were recovered from dental plague samples while the rest were recovered from saliva samples. The pattern of biofilm formation capability of *Streptococcus mutans* test isolates recovered from saliva samples was different from those recovered from dental plaque. Testing forty isolates recovered from each of saliva and dental plaque specimens showed that 26, 12 and 2 of saliva isolates were strong, moderate and weak biofilm producers versus 35, 4 and 1 for dental plaque isolates (Fig. 4).

The number and codes of isolates showing strong, moderate and weak biofilm production of *S. mutans* recovered from dental plaque and saliva samples as determined by microtiter plate are shown in Table 3.

**Table 3:** Numbers and codes of *S. mutans* isolates recovered from dental plaque and saliva samples showing strong, moderate and weak biofilm production as determined by microtiter plate

Biofilm formation degree	Numbers and codes () of isolates recovered from:	
	Dental plaque samples	Saliva samples
Strong	35 (dp1,3,5,8,9,10,11,12,13,14,15,16,17,18,19,20,21,22,23,24,25,26,28,29,30,31,32,33,34,35,37,40,41, 45, 50)	26 (ss1,2,3,5,6,9,10,11,12,13,14,15,16,18,20,22,23,24,29,30,32,33,38,40,41,44,)
Moderate	4 (dp2,6,7,27)	12 (ss17, 19, 21, 25, 26, 31,34,35,36,37,39,42)
Weak	1 (dp4)	2 (ss27, 28)

Some isolates showing different biofilm formation degrees were selected for testing antibiofilm activity of green tea extracts.

### Determination of antibiofilm activity of green tea extracts against some selected *S. mutans* isolates

Aqueous and alcoholic green tea extracts were assayed for their antibiofilm activity using 20 *Streptococcus mutans* isolates, which included 10 recovered from saliva and other 10 from dental plaque specimens. The selected isolates from saliva samples included 7 strong (2 ss (CCASU-SM 25), 5 ss, 13 ss, 14 ss, 24 ss, 30 ss and 40 ss), 1 moderate (21 ss), 2 weak (27 ss, 28 ss) while the selected isolates from dp samples included 6 strong (dp1, dp16, dp25, dp30 (CCASU-SM26), dp35 and 37dp), 3 moderate (dp 2, dp 6, dp 27), 1 weak (dp 4).The activity was measured by determining minimum biofilm inhibitory concentration (MBIC) for each extract against the test isolates and the results are depicted in Table 4.

**Table 4:** Antibiofilm activity of alcoholic and aqueous green tea extracts against 20 *Streptococcus mutans* isolates recovered from dental plaque and saliva specimens (10 isolates each)

Biofilm formation degree	Isolate code	MBIC (mg/ml) of green tea extracts	
		Aqueous extract	Alcoholic extract
Strong	ss 2 (CCASU-SM 25)	25 ± 0.026	6.25 ± 0.020
	ss 5	25 ± 0.029	6.25 ± 0.035
	ss 13	12.5 ± 0.153	6.25 ± 0.045
	ss 14	50 ± 0.1	6.25 ± 0.050
	ss 24	25 ± 0.173	12.5 ± 0.040
	ss 30	50 ± 0.11	12.5 ± 0.055
	ss 40	6.25 ± 0.029	3.125 ± 0.050
	dp 1	25 ± 0.1	12.5 ± 0.032
	dp 16	12.5 ± 0.098	6.25 ± 0.045
	dp 25	25 ± 0.11	6.25 ± 0.088
	dp 30 (CCASU-SM 26)	50 ± 0.091	12.5 ± 0.02
	dp 35	6.25 ± 0.161	3.125 ± 0.25
	dp 37	50 ± 0.11	12.5 ± 0.02
Moderate	ss 21	12.5 ± 0.068	6.25 ± 0.09
	dp 2	25 ± 0.05	12.5 ± 0.05
	dp 6	25 ± 0.1	6.25 ± 0.03
	dp 27	12.5 ± 0.195	12.5 ± 0.1
Weak	ss 27	6.25 ± 0.147	6.25 ± 0.07
	ss 28	6.25 ± 0.053	3.125 ± 0.04
	dp 4	6.25 ± 0.06	3.125 ± 0.11

^*^The activity was expressed as minimum biofilm inhibitory concentration (MBIC)

## Discussion

Dental caries is one of the most common diseases affecting the oral cavity which is mostly caused by the facultative anaerobic bacteria, *Streptococcus mutans*. Its ability to form biofilm and to provide adhesion of microorganism to each other and to the tooth enamel, play an important role in developing dental caries which could further lead to serious condition of infective endocarditis. So, preventing this disease was and still one of the most challenging problems in the oral dental practice. Its main pathogenicity is related to its ability to increase resistance against different antimicrobial agents and to be less phagocytized by immune cells (Gurenlian 2015). The adherence and attachment of *S. mutans* to tooth enamel is mainly due to its ability to synthesize glucan, the building block of polysaccharide matrix from sucrose that increases the efficacy of adhesion and attachment. *S. mutans* possess glucosyltransferase (GTF) enzyme that converts the sucrose to fructose and glucose which is added to growing polymer of glucan forming growing exopolysaccharide matrix. The cariogenic bacteria encodes three genes of glucosyltransferase enzymes (gtfs) (gtfB, gtfC, gtfD). GTFB and GTFC enzymes synthesize water-insoluble glucans which are rich in -1,3-glucosidic linkages while GTFD, produces water-soluble glucans which are rich in -1,6-glucosidic linkages enhancing the coherence of microorganism to each other and adherence to the tooth enamel (Lu et al. 2019; Matsumoto-Nakano 2018). The glucan enhances adhesion of *S. mutans* by forming hydrogen bond to salivary pellicle and other bacteria increasing the biofilm resistance to different chemotherapeutic agents and the host defense (Banas 2004). Also, another component of *S. mutans* mediating binding the microorganism to glucan is glucan binding protein (Gbp) composing of 4 types Gbp A, B, C and D. The GbpC and GbpB are associated with the bacterial cell wall of *S. mutans* acting as a specific receptor for glucan which plays a role in microorganism adhesion and biofilm formation (Krzyściak et al. 2014).

In this study, tube and microtiter plate methods were used to detect and quantify biofilm formation of *Streptococcu*s test isolates. A total number of 150 *Streptococcus* test isolates were tested for their biofilm capability as determined by tube method. About 100 test isolates (66.67%) were found to be biofilm producers and 50 test isolates (33.34%) showed no biofilm production. The 100 test isolates showed to be biofilm producers were identified as *Streptrococcus mutans* while the rest 50 test isolates were recognized as *S. salivarius* (Steiner-Oliveira et al. 2007). According to the results shown by tube method all the 50 tested isolates which did not show any biofilm production activity were identified as *S. salivarius* and this may be due to that *S. salivarius* is a non-biofilm forming bacteria and does not communicate with other microorganisms incorporated in biofilm matrix on the tooth enamel. The biofilm inhibitors produced by *S. salivarius* such as; fructosyltransferase (FTF) and exo-beta-D-fructosidase (FruA) which are highly secreted in medium of high concentration of sucrose has a role in biofilm inhibition. As, FruA cleavages sucrose and inhibits the production of glucan which contributes mainly in biofilm development (Steiner-Oliveira et al. 2007; Ogawa et al., 2011). Also, the results of our study were found to be in accordance with another study performed by Pita et al. comparing between biofilm formation between *S. mutans* and *S.* salivarius. Pita et al. stated that among *S. mutans* and *S. salivarius* in biofilm formation and development, *S. salivarius* exhibited the lowest capacity to form biofilm compared to *S. mutans* (Bidossi et al. 2018; Pita et al. 2015)*.*

The test isolates of *S. mutans* (100) were further screened for their biofilm production by microtiter plate method as this method provides an accurate measurement of biofilm formation by microorganisms. As determined by the microtiter plate, it was observed that the dental plaque samples have the ability to form higher quantities of strong biofilm pattern compared to those of saliva specimens, this is evidenced by the strong biofilm production by 35 isolates recovered from dental plaque compared to only 26 isolates recovered from saliva. Also, 4 test isolates recovered from dental plaque (5%) showed moderate biofilm production capability versus that of 12 test isolates recovered from saliva specimens (15%) and 1 test isolate recovered from dental plaque (1.25%) showed weak biofilm production capability in comparison to 2 test isolates recovered from saliva sample (2.5%). Comparing the data obtained from samples recovered from dental plaque and saliva samples it was observed that isolates recovered from dental plaque have the ability to form stronger biofilm than those recovered from saliva samples. This may be related to the low concentration of *S. mutans* in saliva samples (Al-mudallal et al. 2008; Banas 2004). The results showed that 35 *Streptococcus* test isolates recovered from dental plaque (43.75%) were strong biofilm producers while the same corresponding character degree was exhibited by only 26 *Streptococcus* test isolates recovered from saliva (32.5%). *Leal* and *Mickenautsch* reported that this can be attributed to that the percentage of *S. mutans* in dental plaque was higher than that of saliva which consequently may be due to the variability of sampling method in saliva and dental plaque specimens and this can affect the pattern of formed biofilm (Leal and Mickenautsch 2010). The prevalence of biofilm production among dental plaque isolates as compared to those isolated from saliva isolates could be due to dental plaque constituted an immobilized surface for adherence of microbial community while saliva represents mobilizing viscous solution that could be of low support for biofilm formation. Also, dental plaque represents deposits of debris which act as a substrate for glucan formation by *Streptococcus* species (Hsu et al. 2010; Motisukiet al. 2005). Saliva contains enzymes such as lysozyme that act adversely on the microbial community (Al-mudallal et al. 2008; Steiner-Oliveira et al. 2007). The antimicrobial mechanism of lysozyme is related to its enzymatic activity in the bacterial peptidoglycan where it hydrolyses β-1,4 glycosidic bonds between N-acetylglucosamine and N-acetylmuramic acid and because of its cationic and hydrophobic properties, lysozyme has the ability to aggregate oral bacteria affecting their adherence to the tooth enamel, distributing the biofilm structure and enhancing clearance (Hukić et al. 2018).

Different treatment prescriptive in dental caries for biofilm inhibition have been investigated such as chlorhexidine as one of the first antiseptic agents used for dental caries. It has plaque inhibitory activity and blocks the activity of acidic glycoprotein present in saliva, thus decreases the tooth plaque adhesion but it was reported to have serious side effects such as vomiting, diarrhea and tooth discoloration. Quaternary ammonium salts have proposed to be used as antibiofilm agents as the positive charged quaternary ammonium moieties bind to negatively charged bacteria, thus disturb their nature balance. However, these agents have side effects such as coma, convulsions and hypotensive actions which limit their use. Cetylpyridinium chloride has proven to have antibacterial activity by affecting the cell membrane of bacteria decreasing the biofilm development and also polyvinylpyrrolindine inhibits biofilm formation by penetrating the bacterial membrane and inhibiting the metabolism of protein, fatty acid and nucleotides of bacterial cell. Fluoride inhibits enolase enzyme affecting bacterial growth adversely and reduces acid production of *S. mutans*. Long term administration of this agent at high concentration can lead to dental and skeletal fluorosis, thus its use should be reconsidered (Razuqi et al. 2012; Hengge 2019).

The continuous search for alternatives other than the present antibiofilm agents became a required demand because of the high economic cost of the present used synesthetic antibiofilm agents and the possibility of developing serious side effects upon their long term administration (Qiu et al. 2020). The use of herbal products has a low-cost value, least probability of developing major side effects comparable to the conventional antibiofilm agents that are available. So, in this study two extracts from the herbal green tea plant (*Camellia sinensis*) were checked for their antibiofilm activity (Lu et al. 2019).

Green tea is known to have potential of antibacterial activity. The health-promoting effects of green tea are mainly attributed to its polyphenol contents commonly referred to as catechins. There are four main types of catechins: epigallocatechin-3- gallate (EGCG), epigallocatechin, epicatechin-3- gallate and epicatechin (Faraz et al. 2012). Epigallocatechin-3- gallate (EGCG) exhibits anti-biofilm activity mainly by reducing the adherence and attachment of *S. mutans* to the surface of tooth enamel, interfering with bacterial glucosyltransferases (GTF) through reduction of the expression of three genes (*gtfB*, *gtfC*, *gtfD*) that encode these enzymes. Glycosyltransferases enzymes are responsible for converting sucrose to glucan, the building block of the biofilm-associated exopolysaccharide matrix thus inhibiting the biofilm formation (Hengge 2019). Also, another study demonstrated that the green tea may have indirect antibacterial activity by acting on protective saliva components such as secretory immunoglobulins, lysozyme, lactoferrin, oral peroxidases, histatins and mucins (Salama and Alsughier 2019; Taylor et al. 2005). In other recent studies, biofilm formation was suggested to be involved in intercellular cell to cell communication to each other by quorum sensing, EGCG may disturb signaling via autoinducer-1/LuxR-dependent or autoinducer-2-dependent quorum sensing systems (Hengge, 2019; Miquel et al. 2016).

The antibiofilm activity of aqueous and alcoholic green tea extracts was evaluated by microtiter plate assay against some selected test isolates of *Streptococcus mutans* collected and recovered from saliva and dental plaque specimens. The results showed that aqueous and alcoholic green tea extracts have antibiofilm activity against *Streptococcus mutans*.

The prepared alcoholic green tea extract was observed to have antibiofilm activity at lower concentration than that of aqueous extract. This could be due to its content of more active ingredients, alcoholic extract may have more content of the active constituents epicatechin and epigallocatechin-3- gallate (EGCG) than that of aqueous extract and as it appears from several studies, the antibacterial action of green tea is due to the polyphenolic components (Mageed and Saliem 2015; Blumberg et al. 2015).

It was also observed that alcoholic extract of green tea exhibited antibiofilm activity against the strong biofilm producer isolates at concentrations 3.1, 6.3, 12.5 mg/ml and the aqueous extract of green tea exhibited antibiofilm activity at concentrations 6.3, 12.5, 25 and 50 mg/ml.

From this study, it can be concluded that the variability in sampling method in dental plaque and saliva samples may affect pattern of biofilm producing bacterial species. *Streptococcus mutans* species were shown to be biofilm producers in contrast to that of *Streptococcus salivarius* which showed no biofilm formation activity. Green tea can be used for dental caries prevention as it is available and of low economic cost. Both alcoholic and aqueous extracts have antibiofilm activity against the cariogenic bacteria *Streptococcus mutans*. Thus, these extracts can be used for preparation of antibiofilm dental formulas to be applied for therapeutic purposes or in oral hygiene practice. The alcoholic extract proved to have antibiofilm activity at lower concentrations than that of aqueous extract indicating the extraction of more types and/or amounts of components with antibiofilm activity.

## Data Availability

The original data and the datasets supporting the conclusions of this current study were included within the article, any required explanation and help is available through the corresponding author.

## Abbreviations

*S. mutans*: *Streptococcus mutans*
*S. salivarius*: *Streptococcus salivarius*
MBIC: Minimum biofilm inhibitory concentration
dp: Dental plaque
ss: Saliva sample
EC: Epicatechin
EGC: Epigallocatechin
ECG: Epicatechin gallate
EGCG: Epigallocatechin gallate
OD: Optical density
TM: Test method
MTP: Microtiter plate
GTF: Glucosyltransferase
Gbp: Glucan binding protein
FTF: Fructosyltransferase
FruA: Exo-beta-D-fructosidase
VP: Acetoin production
ESC: B- Glucosidase hydrolysis
PYRA: Pyrrolidinyl arylamidase
αGAL: α-Galactosidase
ßGUR: ß-Glucuronidase
ßGAL: ß-Galactosidase
PAL: Alkaline phosphatase
LAP: Leucine aminopeptidase
ADH: Arginine dihydrolase
RIB: d-Ribose
ARA: l-Arabinose
MAN: d-Mannitol
SOR: d-Sorbitol
LAC: d-Lactose
TRE: d-Trehalose
INU: Inulin
RAF: d-Raffinose
AMD: Starch
GLYG: Glycogen

## References

[CR1] Acumedia (2011) Mitis Salivarius Agar. 4–5

[CR2] Al-Mudallal N, Al-Jumaily E, Muhimen N, Al-Shaiban A (2008). Isolation and Identification of *mutan’S Streptococci*. ANJS.

[CR3] Banas A (2004). Virulence properties of *Streptococcus mutans*. Front Biosci.

[CR4] Banu L (2010). Gene expression in *Streptococcus Mutans* biofilms.

[CR5] Bidossi A, De Grandi R, Toscano M, Bottagisio M, De Vecchi E, Gelardi M, Drago L (2018). Probiotics *Streptococcus salivarius* 24SMB and *Streptococcus oralis* 89a Interfere with biofilm formation of pathogens of the upper respiratory tract. BMC Infect Dis.

[CR6] Blumberg J, Bolling B, Chen C, Xiao H (2015). Review and perspective on the composition and safety of green tea extracts. EJNFS.

[CR7] Fajriani, Sartini S, Handayani H, Putri D (2020) The role of green tea extract on inhibiting porphyromonas gingivalis as a major periodontitis pathogen : in vitro study. Sys Rev Pharm 2020;11(8):152–155

[CR8] Faraz N, Islam Z, Rehman R and Sehrish (2012) Antibiofilm forming activity of naturally occurring compound. J Biomedica, vol. 171–75

[CR9] Hengge R (2019). Targeting bacterial biofilms by the green tea polyphenol EGCG. J Molecules.

[CR10] Hsu K, Osgood R, Cutter G, Childers N (2010). Variability of two plaque sampling methods in quantitation of *Streptococcus mutans*. J Caries Res.

[CR11] Hukić M, Seljmo D, Ramovic A, Ibrisimovic M, Dogan S, Hukić J, Bojic E (2018). The effect of lysozyme on reducing biofilms by *Staphylococcus aureus*, *Pseudomonas aeruginosa*, and Gardnerella vaginalis: an in vitro examination. J Microb Drug Resist.

[CR12] Ito Y, Ito T, Yamashiro K, Mineshiba F, Hirai K, Omori K, Yamamoto T, Takashiba S (2020). Antimicrobial and antibiofilm effects of abietic acid on cariogenic *Streptococcus mutans*. J Odontology.

[CR13] Gurenlian JR (2015). American dental hygienists’ association: the role of dental plaque biofilm in oral health. J Dent Hyg.

[CR14] Krzyściak W, Jurczak A, Kościelniak D, Bystrowska B, Skalniak A (2014). The virulence of *Streptococcus mutans* and the ability to form biofilms. Eur J Clin Microbiol Infect Dis.

[CR15] Kwasny S, Opperman T (2010). Static biofilm cultures of gram positive. Curr Protoc Pharmacol.

[CR16] Leal S, Mickenautsch S (2010). Salivary *Streptococcus mutans* count and caries outcome-a systematic review. J Minim Interv Dent.

[CR17] Lu L, Hu W, Tian Z, Yuan D, Yi G, Zhou Y, Cheng Q, Zhu J, Li M (2019). Developing natural products as potential anti-biofilm agents. J Chin Med.

[CR18] Mageed M, Saif S (2015). Antibacterial effects of green tea extracts on *Aggregatibacter actinomycetemcomitans* : In vitro study. JBCD.

[CR19] Mathur T, Singhal S, Khan S, Upadhyay FT, Rattan A (2006). Detection of biofilm formation among the clinical isolates of *Staphylococci*: an evaluation of three different screening methods. Indian J Med Microbiol.

[CR20] Matsumoto-Nakano M (2018). Role of *Streptococcus mutans* surface proteins for biofilm formation. Jpn Dent Sci Rev.

[CR21] Mehrishi P, Agarwal P, Broor S, Sharma A (2020). Antibacterial and Antibiofilm properties of medicinal plant extracts against multi drug resistant *Staphylococcus* species and non fermenter bacteria. J Pure Appl Microbiol.

[CR22] Miquel S, Lagrafeuille R, Souweine B, Forestier C (2016). Anti-biofilm activity as a health issue. Front Microbiol.

[CR23] Mohamed A, Rajaa A, Khalid Z, Fouad M, Naima R (2013). Comparison of three methods for the detection of biofilm formation by clinical isolates of *Staphylococcus aureus* isolated in casablanca. IJSR.

[CR24] Motisuki C, Lima L, Spolidorio D, Santos-Pinto L (2005). Influence of sample type and collection method on *Streptococcus mutans* and *Lactobacillus* Spp. counts in the oral cavity. Arch Oral Bio.

[CR25] Ogawa A, Furukawa S, Fujita S, Mitobe J, Kawarai T, Narisawa N, Sekizuka T, Kuroda M, Ochiai K, Ogihara H, Kosono S, Yoneda S, Watanabe H, Morinaga Y, Uematsu H, Senpuku H (2011). Inhibition of *Streptococcus mutans* biofilm formation by*Streptococcus salivarius* FruA. Appl Envivon Microbiol.

[CR26] Pita P, Rodrigues J, Ota-Tsuzuki C, Miato T, Zenobio E, Giro G, Figueiredo L, Gonçalves C, Gehrke S, Cassoni A, Shibli J (2015). Oral *Streptococci* biofilm formation on different implant surface topographies. Biomed Res Int.

[CR27] Qiu W, Zhou Y, Li Z, Huang T, Xiao Y, Cheng L, Peng X, Zhang L, Ren B (2020). Application of antibiotics/antimicrobial agents on dental caries. Biomed Res Int.

[CR28] Razuqi N, Dhahir S, Ascar E (2012). Biological effect of aqueous & alcoholic extracts of green tea leaves of some pathogenic bacteria in vitro. J Kufa for Chem.

[CR29] Rolim W, Lamilla C, Pieretti J, Diaz M, Tortella G, Diez M, Barrientos L, Seabra A, Rubilar O (2019). Comparison of antibacterial and antibiofilm activities of biologically synthesized silver nanoparticles against several bacterial strains of medical interest. J Energ Ecol Environ.

[CR30] Salama M, Alsughier Z (2019). Effect of green tea extract mouthwash on salivary *Streptococcus mutans* counts in a group of preschool children: an in vivo study. Int J Clin Pediatr Dent.

[CR31] Steiner-Oliveira C, Maciel F, Rodrigues L, Napimoga M, Pimenta L, Höfling J, Gonçalves R (2007). An in vitro microbial model for producing caries-like lesions on enamel. Braz J Oral Sci.

[CR32] Taylor P, Hamilton-Miller J, Stapleton P (2005). Antimicrobial properties of green tea catechins. Food Sci Technol Bull.

[CR33] Wang Y, Shen X, Ma S, Guo Q, Zhang W, Cheng L, Ding L, Xu Z, Jianga J, Gao L (2020). Oral biofilm elimination by combining iron-based nanozymes and hydrogen peroxide-producing bacteria. Biomater Sci.

